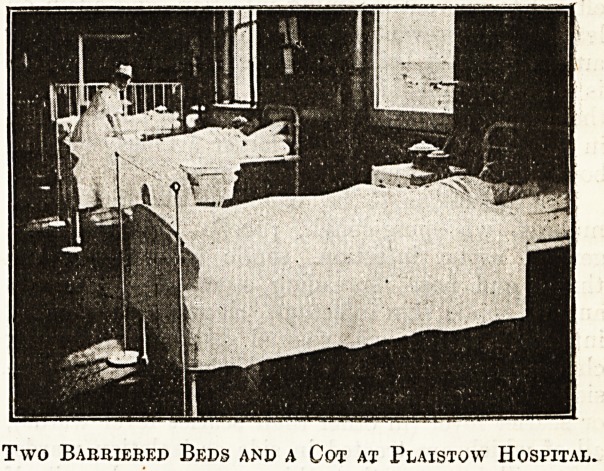# Preventive Methods in Provincial Institutions

**Published:** 1912-08-17

**Authors:** A. Knyvett Gordon

**Affiliations:** formerly Medical Superintendent of Monsall Hospital and Lecturer on Infectious Diseases in the University of Manchester.


### SOME FEVER HOSPITALS AND THEIR WORK.*

### IV.—Preventive Methods in Provincial Institutions.

By A. KNYVETT GORDON, M.B. Cantab., formerly Medical Superintendent of Monsall Hospital and Lecturer on Infectious Diseases in the University of Manchester.

In considering the question of the best method of preventing the infection of one patient by another, we have sooner or later to face the problem of local treatment of the throat and nose, for on this hangs much of the comparative success of the modern fever hospital.

Here, again, we have to compromise. In a private house, where there is only one patient, we have only one kind of infection to consider—namely, that from the patient's own organisms. Consequently, we can adapt the local treatment exactly to the clinical condition, and do as much or as little douching, swabbing, spraying as we fancy.

In hospital, however, we have to remember that, broadly speaking, any one patient (with scarlet fever) may infect any other. Some cases are more likely to do this than others, and we label them "septic" accordingly, but further we cannot go.

### A Misnomer for a Bad Method.

Consequently, we have to be very careful that in the attempt to cure one throat we do not carry infection to another. Formerly, as I pointed out, this did not receive the attention it deserved, and it was customary for a nurse to irrigate the throat of one patient with a Higginson's syringe and then to proceed to do the same to the next, and so on all round the ward, the only precaution taken being the dipping of the nozzle of the syringe momentarily into an "antiseptic" solution between the treatment of each patient, so that under this *régime* post-scarlatinal diphtheria was regarded as a "complication" of scarlet fever!

Nowadays we have broadly two schools; some hospital superintendents hold that there should be as little throat treatment as possible in order to minimise the amount of infective material which it is possible for a nurse to carry about with her; some also believe that irrigation of the fauces tends to increase the incidence of otorrhœa.

Others hold that irrigation of the throat and nose tends not only towards the healing of the inflamed surfaces—in other words, that it is best for the patient's own infection—but that it removes alien infection which the patient may be incubating in these parts. But they endeavour to make the treatment safe from conveying infection to others.

As regards the details of the latter method, I may, perhaps, be permitted to refer to my own practice when in charge of Monsall Hospital, full details of which were given in an article in the special number of the *Practitioner* which dealt with scarlet fever and diphtheria. The douche-can is carried on a metal and glass trolley from bed to bed. On the trolley is also a bowl containing recently boiled nozzles—two for each patient—immersed in a solution of boric acid, and another with recently boiled swabs. The douche-can contains sterile water or normal saline solution, and there is a receptacle below containing Izal solution for the reception of the dirty nozzles and swabs.

The nurse starts by scrubbing her hands with antiseptic soft soap, and then swabbing them with turpentine followed by methylated spirit. She then puts on sterile rubber gloves, which she does not remove until the end of her round. A separate nozzle is used for the throat and nose, and the nurse cleanses the gloved hands between the treatment of each patient by holding them under running water from the tap for one minute. The height of the douche-can is fixed so that it cannot deliver a too forcible jet. The chief objection to the douche—namely, that it tends to produce otorrhœa—is met by the fact that in a long series of cases treated

Two Barriered Beds and a Cot at Plaistow Hospital.

with and without irrigation respectively I found the incidence of otorrhœa lower in those patients that had been irrigated. I believe, however, that the forcible jet from a Higginson's or rubber-ball syringe may produce otorrhœa. My own view is that no method of treatment is quite safe unless rubber gloves are used by the nurse. The only objection is their expense.

### The Barrier System at Plaistow Hospital.

It is, however, also necessary to guard against infection by means of utensils other than those for the treatment of throats, and this has led to the system of barriers. This has, as far as I know, reached its highest development at Plaistow Hospital under the direction of Dr. J. Biernacki, to whose courtesy I am indebted for some notes, and also for the photograph from which the illustration is produced which shows two beds and a cot barriered, and the sister sterilising her hands within the barrier after attending to a patient. Dr. Biernacki does not use screens, but merely a cord placed at the foot of each bed to be barriered.

* Previous articles appeared in The Hospital of July 13, 20, and Aug. 10.

Each barriered bed has a locker to itself which contains the following articles:—Washing blanket, soap in dish, methylated spirit, cloth, knife, spoon, and fork, airtight aseptic box for clean dressings, three lotion bowls, two receivers, metal swab holder, throat syringe, ear syringe, dressing forceps, washing flannel and towel, toilet bag for head, fomentation wringer, apparatus for nasal and rectal feeding and giving enemas (in separate covered bowls). On the bed cardboard is a clinical thermometer in a vial. On a glass wall bracket are: Spray, measure, and towel for giving medicines, medicine bottles, jars for ointment and powder. At the bed head is a holland bag for toys, etc.

In the ward kitchen is a stock of sterilised feeders, mugs, plates, etc. Any of these articles taken within a barrier must be sterilised on removal from it. Within the barrier are also bowls containing lotion for "sterilising" the nurses' hands. Only articles actually required for the treatment of the patient are kept within the barrier at the time.

The nurse on entering a barrier places her hands in the bowl of lotion (the arms being bare to the elbow) to prevent incoming infection, and again on leaving it to prevent outgoing infection. All articles are sterilised by boiling in a special steriliser, which is heated by the high-pressure steam that supplies the radiators in the ward. There is a large trough in the sink room in which larger articles, such as bowls, can be sterilised by a steam coil.

The infections to be barriered are diphtheria, mumps, whooping-cough, rubella, typhoid fever, general septic infection, septic infection of the throat and nose, spreading stomatitis, Vincent's angina, septic skin infections, ringworm, spreading infections of the eye, vaginal discharge in young children, syphilis. But at Plaistow it is not considered advisable to barrier chicken-pox, measles, or scarlet fever (in other than scarlet fever wards), all of which are treated in side or isolation wards.

It will be seen that this system resolves itself into a very careful scheme for protection of utensils, and of asepsis as regards the nursing; and it absolutely ignores the possibility of infection by aërial convection for any one of the barriered diseases. In the 3 others aërial convection is thought possible.

At Monsall, each barriered bed was surrounded by screens on which were hung sheets wet with a weak solution of Izal, and my own experience was that it was safe to barrier the last three diseases also, probably because the screens stopped what little aërial convection there was as far as the patient in the next bed was concerned. I do not believe in aërial convection to the other end of the ward.

At Plaistow the barriered beds are at one end of the ward only, so that the patients who are not barriered can come and go without crossing the barriered part. There are never more than four barriered beds in a ward, and generally only three. The results at Plaistow have been practically perfect, and it would be difficult to find a hospital so well equipped as regards appliances for securing asepsis, or where the technique of the nursing has been so well thought out. The buildings, incidentally, are modern and were designed by Mr. E. T. Hall; they somewhat resemble the pavilions in another of his hospitals, the Park Hospital under the Asylums Board. I should add that there is one great disadvantage of a barrier of screens—namely, that the nurse cannot see the patient from a distance in the ward. At Monsall, however, a cot net was employed in the case of young children.

Methods at the Edinburgh City Hospital.

We now come to another modern hospital—namely, that for the City of Edinburgh. This is a very large establishment, and consists of 600 beds distributed in exceptionally roomy wards over a very large area. Structurally the wards are magnificent, and even elaborate. Inasmuch as each pavilion contains four side rooms, or separation wards, and there are isolation blocks as well, it has not been found necessary to adopt the barrier system, but the septic and doubtful cases are nursed in a side room by the ordinary staff of the ward. The nurse, however, wears an overall when attending to any such patient, and separate throat instruments and crockery are provided for each side ward. There is a special pavilion for patients in whose throats diphtheria bacilli have been found.

The number of different diseases treated is great, practically all the common infections being admitted; this is made possible by the number of separate pavilions. Thus, in addition to scarlet fever, diphtheria, and enteric fever, cases of measles, whooping-cough, erysipelas, puerperal fever, and phthisis are normally provided for. Throat treatment in scarlet fever wards is reserved for septic cases only, which are irrigated with a douche-can or a syringe.

Only "clean" cases, that is to say, those in whom there is no discharge from nose, ears, or wounds, are sent to the convalescent wards for scarlet fever, and all patents have to spend at least three weeks in the acute ward; if a patient develops a mucous discharge in a convalescent ward he is placed in the side room attached to that ward.

On discharge, either a disinfecting bath is given in the discharging house, followed by a night spent in a special ward kept for this purpose, or, after disinfection, as before in the discharging house, the patient spends a full week in quarantine in a special pavilion. This latter plan is excellent, but very few hospitals are sufficiently well equipped with separate pavilions to enable it to be carried out. Many of the isolation pavilions at Edinburgh are on the cottage plan, containing four beds each. These are used mainly for combinations of infectious diseases, such as scarlet fever and measles, or chicken-pox. Structurally this plan is ideal, but it is only possible with a large number of nurses, and very few authorities provide sufficient to work this system.

Sun rooms have been used mainly for the treatment of severe cases in the open air, or under partial shelter according to the state of the weather, a method which the Medical Superintendent, Dr. C. B. Ker—to whose courtesy I am indebted for much information about the hospital—has found to give excellent results, especially, as I understand, in severe cases of whooping-cough.

## Figures and Tables

**Figure f1:**